# The impact of tumor board on cancer care: evidence from an umbrella review

**DOI:** 10.1186/s12913-020-4930-3

**Published:** 2020-01-31

**Authors:** Maria Lucia Specchia, Emanuela Maria Frisicale, Elettra Carini, Andrea Di Pilla, Danila Cappa, Andrea Barbara, Walter Ricciardi, Gianfranco Damiani

**Affiliations:** 1 Fondazione Policlinico Universitario A. Gemelli IRCCS, Largo A. Gemelli 8, 00168 Rome, Italy; 2 Università Cattolica del Sacro Cuore, Rome, Italy; 3 Local Health Authority, ASL ROMA 1, Rome, Italy

**Keywords:** Healthcare, Tumor board, Multidisciplinary team, Diagnostic accuracy, Personalized treatment, Personalized medical care, Teleconsultation

## Abstract

**Background:**

Tumor Boards (TBs) are Multidisciplinary Team (MDT) meetings in which different specialists work together closely sharing clinical decisions in cancer care. The composition is variable, depending on the type of tumor discussed. As an organizational tool, MDTs are thought to optimize patient outcomes and to improve care performance. The aim of the study was to perform an umbrella review summarizing the available evidence on the impact of TBs on healthcare outcomes and processes.

**Methods:**

Pubmed and Web of Science databases were investigated along with a search through citations. The only study design included was systematic review. Only reviews published after 1997 concerning TBs and performed in hospital settings were considered. Two researchers synthetized the studies and assessed their quality through the AMSTAR2 tool.

**Results:**

Five systematic reviews published between 2008 and 2017 were retrieved. One review was focused on gastrointestinal cancers and included 16 studies; another one was centered on lung cancer and included 16 studies; the remaining three studies considered a wide range of tumors and included 27, 37 and 51 studies each. The main characteristics about format and members and the definition of TBs were collected. The decisions taken during TBs led to changes in diagnosis (probability to receive a more accurate assessment and staging), treatment (usually more appropriate) and survival (not unanimous improvement shown). Other outcomes less highlighted were quality of life, satisfaction and waiting times.

**Conclusions:**

The study showed that the multidisciplinary approach is the best way to deliver the complex care needed by cancer patients; however, it is a challenge that requires organizational and cultural changes and must be led by competent health managers who can improve teamwork within their organizations. Further studies are needed to reinforce existing literature concerning health outcomes. Evidence on the impact of TBs on clinical practices is still lacking for many aspects of cancer care. Further studies should aim to evaluate the impact on survival rates, quality of life and patient satisfaction. Regular studies should be carried out and new process indicators should be defined to assess the impact and the performance of TBs more consistently.

## Background

Cancer care is a complex path that requires collaboration among professionals with complementary skills who work together to share the latest evidence and to pool their expertise, exchanging information through a regular flow of communication. Technological advances and the possibility to customize patient treatment plans (target, molecular and radiation therapy) have further increased the need for regular interactions between healthcare professionals from various areas of expertise. Consequently, over the last decades, scientific evidence demonstrates that cancer care has been increasingly delivered through multidisciplinary team (MDT) interventions [1].

A MDT is a team composed of professionals from different clinical specialties who work together to make decisions about the recommended clinical pathway of an individual patient [2, 3]. MDT meetings are a fundamental part of a complex care path, during which MDTs gather to discuss on a series of patients in order to achieve a definite staging and formulate a shared treatment plan, in the light of the best available evidence for customized treatment options and appropriate follow-up. In most cases, the multidisciplinary approach could be a great challenge and a useful platform for the coordination of care, and a tool to optimize decision-making and communication processes. It consequently improves the healthcare system and its experience for both patients and professionals, particularly concerning oncological diseases [1, 4, 5].

When MDT meetings are focused on oncological patient’s care, they are called Tumor Boards (TBs) [3]. They could also be addressed as Multidisciplinary Cancer Conferences (MCC), which have been defined by Wright et al., 2007 as forum for healthcare providers aiming to discuss diagnostic and treatment of cancer patients [6]. This term has been used as a synonym of TB [6, 7]; therefore, this review will comprehensively address this concepts as TBs.

While in the literature the multidisciplinary approach in cancer care is described from 1975, it was not regularly implemented in clinical practice until the late 1990’s (more specifically, starting from 1997) [8, 9]. From that moment on, the use of the multidisciplinary approach has continually increased, eventually becoming routine with the constitution and improvement of TBs being pursued as key objectives in many cancer plans and clinical practice guidelines [10–17].

In the past, internists or general practitioners managed oncological patients individually, only seeking counselling from cancer specialists when they deemed it necessary. The formal establishment of multidisciplinary engagement for oncological patients care began with the creation of TBs. In the beginning, TBs aimed to advise and assist the physicians who held the responsibility on clinical management and on care decision. A TB was only called at one point in patient management time, not during the entire staging and treatment pathway, and the patient was rarely present [7].

Over time, TBs have evolved acquiring a more collaborative structure with teams that pay attention to all the aspects of cancer care, including rehabilitation, psychosocial needs and long-term care. The patient could also be present at the meetings and his/her consensus is sought throughout the duration of the treatment process. In addition, treatment decisions and clinical responsibility are shared by the members of the TB. More recently, technological advances have made collaboration among TB members easier by introducing the possibility of “virtual team” meetings when team members are not available in person [7].

The members of TBs and their attendance at meetings depend on several factors which include hospital size and cancer type. In general, professionals eligible to participate as members of the TB are medical and radiation oncologists, surgeons, radiologists, pathologists, nurse specialists, nuclear medicine specialists, palliative medicine physicians, pharmaceutical experts and psycho oncologists. Various professionals with a background in allied health disciplines such as genetics counsellors, nutritionists, plastic surgeons may also be called upon and finally experts specialized in areas related to the tumor site may also be present. Within the TBs, leaders are usually identified for the effective coordination and organization of services and clinical management [7, 18, 19].

Cancer patients can be discussed either in a prospective or retrospective manner. A TB with a prospective approach gathers the collaborating specialists formally at scheduled times in order to review individual cancer patients in a pragmatic way using an evidence-based approach, to discuss diagnosis and formulate future treatment and management plans [3].

The retrospective approach, which will not be addressed by this review, consists of a multidisciplinary discussion of cases with an educational aim, to assess in a multi-professional environment whether the decisions taken for the patient’s management were optimal in an effort to inform and educate the treating physicians in hopes of improving care for future cases [20].

Many studies and integrated reviews have been performed in an effort to evaluate the actual impact that the introduction of TBs have had on the medical practice. The evidence describes aspects of clinical practice such as patient assessment, diagnosis and staging [21], treatment (e.g. adherence to treatment plan or to the guidelines [22–24]) and clinical outcomes (survival, recurrence of cancer, etc.) [12, 25–27]. Although there is awareness that other factors evolved along with the TBs such as innovative technologies in diagnostics and therapy, the scientific research investigated whether the significant investment in time and financial resources for TBs were matched with an effective improvement of the outcomes [4, 5].

This umbrella review aims to provide stronger evidence for healthcare decision-makers by comparing findings from different reviews that address the same topic [28]. The researchers aim to present a comprehensive analysis of the impact of TBs on healthcare outcomes and processes.

## Methods

The study was performed with an umbrella review design which refers to an analysis that compiles evidence from multiple reviews into one document. To evaluate if consistent literature was available, a scoping review was carried out first. Clear objectives/questions were defined accordingly, and detailed inclusion and exclusion criteria were identified. Thereafter, structured search process, quality assessment of the included reviews, and effective data extraction were performed to synthesize the results of the literature review [28].

### Search strategy

A systematic research of reviews was conducted through PubMed and Web of Science databases using the following string: ((cancer OR neoplasm* OR tumour* OR tumor* OR malignanc*) AND (“cancer management” OR “tumor board*” OR “tumour board*” OR “cancer board*” OR “multidisciplinary team*” OR “multidisciplinary meeting*” OR “cancer MDT” OR “cancer care” OR “multidisciplinary conference*” OR “multidisciplinary clinic*” OR “patient care team*” OR “patient care planning”) AND (“clinical decision-making” OR “outcome and process assessment” OR “diagnosis change*” OR survival OR “guidelines adherence” OR morbidity OR mortality OR management)). The investigation of the databases was conducted during January 2019 (until 23rd January). An additional search of citations referenced in the included studies was performed to complete the research. The articles retrieved were screened independently by two researchers by title, then by abstract and eventually by reading the full text according to the inclusion and exclusion criteria outlined in the next paragraph.

### Inclusion / exclusion criteria

PICOS elements (Population, Intervention, Comparator, Outcome, Study type) were used as parameters to define inclusion and exclusion criteria.

(P) The studies selected assessed oncological populations with cancer in different organs. (I) The intervention was defined as “scheduled TBs” in a process of taking in charge patients in a hospital setting. (C) The comparison used was no TBs implementation. (O) Outcomes considered were both clinical and related to care processes. (S) In this study, only systematic reviews were considered. Moreover, other inclusion criteria were: English, French, Spanish, and Italian as language of publication; year of publication later than 1997; and availability of full-texts. We also excluded articles focused only on elderly and pediatric population.

### Selection process and data extraction

The selection of articles followed the criteria defined in the PRISMA Statement and was independently performed by two authors. The same researchers extracted data from the selected reviews. For each review retrieved the main information were categorized in a table and classified as data related to: authors, year and country, databases investigated and date range, number of studies included, quality assessment, study population, aim of the review, outcomes/outputs reported, and main findings.

### Quality assessment

The quality of the reviews retrieved was assessed using the AMSTAR2 tool. This is one of the most widely used instruments to enable a reproducible assessment of the quality of both randomized as well as non-randomized systematic reviews. AMSTAR2 consists of sixteen domains presented in the form of questions. The possible answers are ‘Yes’ if it denotes a positive result; ‘No’ when both the answer is negative or it can’t be provided; ‘Partial Yes’ in case of partial adherence to the standard [29].

The final quality judgment (high, moderate, low, critically low) was performed by two researchers and disagreements were overcome by consensus. The judgement was based on the assessment of specific critical domains, which were identified as the: presence of a protocol registered before the commencement of the review; adequacy of the literature search; justification for excluding individual studies; evaluation of the risk of bias of the studies included; appropriateness of meta-analytic methods when applicable; consideration of risk of bias when interpreting the results of the review; assessment of presence and likely impact of publication bias.

## Results

**Fig. 1:**
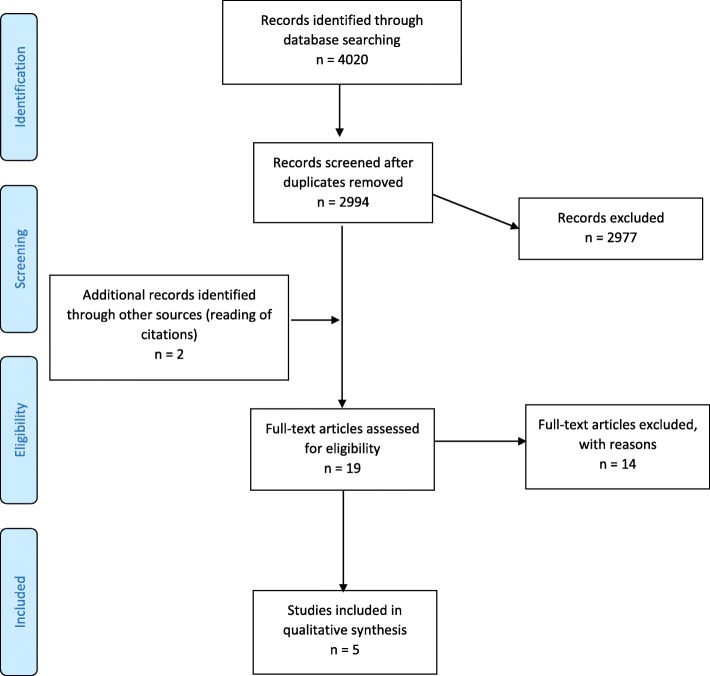
PRISMA Flow chart

A total number of 4020 records were found. After removing duplicates and reading titles and abstracts, five systematic reviews met the inclusion and exclusion criteria. Details about the process of exclusion of records are provided in the PRISMA chart (Fig. 1).

The objectives of four reviews fully aligned with the aim of this umbrella review; all five were focused on assessing the impact and effectiveness of case discussion among TB members on various healthcare outcomes/outputs and processes with reference to both patients and professionals. The review of Lamb et al. (2011) addressed factors which enhanced or impeded effective decision-making in TBs. It also provided an analysis of the impact of TBs that aligned with the objectives of this study. The five reviews ranged from 2008 to 2017. Three of the reviews (Lamb et al., 2011 [30], Prades et al., 2015 [31], Pillay et al., 2016 [32]) considered a wide range of tumors discussed by TBs, while the other two studies were focused on lung (Coory et al., 2008) [33] and gastrointestinal (Basta et al., 2017) [34] cancer discussion. The main characteristics of the studies are summarized in Table 1.

**Table 1:** Characteristics of the reviews included

Authors, year, country	Databases and date range	Number of studies (studies quality)	Cancer types and population	Objective	Outcomes / outputs	Main results
Coory et al., 2008 [38] (Australia)	Ovid Medline, and snowball search. 1984 - July 2007.	16 (studies quality: not assessed)	Lung cancer (both SCLC and NSCLC)	To evaluate and critically appraise the effectiveness of multidisciplinary teams to treat lung cancer and particularly to assess if the TBs, compared to traditional models of care, improves survival, and other outcomes such as practice patterns and waiting times.	Survival. Practice patterns. Waiting times. Satisfaction with care. Visits to GPs. Quality of life.	Weak evidence of a causal association in survival improvement. Stronger evidence of the effect of TBs on changing patient management: increase in the percentage of patients undergoing surgical resection and in the percentage of patients undergoing chemotherapy or radiotherapy with curative intent. Reduced waiting times. Improved patient satisfaction. Reduced number of visits to GPs. No-statistically significant differences in patients’ quality of life.
Lamb et al., 2011 [30] (UK)	Embase, Medline, PsycINFO (using OvidSP), Cochrane database. 1999 - 15th May 2009	37 (studies quality: low to medium)	Breast, lung, gynaecology, urology, upper GI, colorectal, sarcoma, brain, head and neck cancer	To examine the literature on care management decisions in cancer TBs and assess the factors that enhance or impede effective decision-making	Diagnosis. Care management decisions. Adherence to guidelines. Treatment. Implementation of TB decisions. Survival.	Improvement in diagnostic accuracy. Changes in care management decisions (2–52% of cases). Improved adherence to clinical guidelines. More likelihood of patients being offered chemotherapy (7–23%). TB decisions not implemented in 1–16% of cases. Significant increase in survival for patients being offered chemotherapy (3.2–6.6 months).
Prades et al., 2015 [31] (Spain and Belgium)	Medline database. November 2005–June 2012	51 (studies quality: not assessed)	Urological, pancreatic, rectal, head and neck, melanoma, oesophageal, prostate and genitourinary, colon, lung, breast, oesophageal, osteological, skin, gynaecological, and neurological cancer and bone metastases	To assess the impact of TBs on patient outcomes in cancer care and identify their objectives, organisation and ability to engage patients in the care process.	Diagnosis and/or treatment planning. Survival. Patient quality of life. Patient and clinician satisfaction. Waiting times. Care coordination for professionals and patients.	Improvement in diagnosis and staging accuracy; more appropriate treatment through preoperative review of imaging and pathology results; more up-to-date treatment; structured follow-up care plan. Improved survival. Patient quality of life improvement. Improved patient and clinician satisfaction. Reduced waiting times. Coordination and continuity of care improvement by reducing time from diagnosis to treatment; achieving early and appropriate referral patterns. Furthermore: teaching environment for healthcare professionals and junior doctors; increased enrolment in tumour registry; maintaining a commitment to research and clinical trials
Pillay et al., 2016 [32] (Australia)	OVID Medline, PsycINFO, and EMBASE databases. 1995 - April 2015	27 (studies quality: not assessed)	Colon or rectal, lung, oesophageal or gastric, urological, gynaecological, breast, hematologic and head and neck tumours. Mean or median age range: 54–71 years. Sample size range: 47–6760.	To summarise, integrate, and critically evaluate the literature regarding the impact of TBs on patient assessment, diagnosis, management and outcomes in oncology settings.	Patient assessment/diagnosis. Patient management/clinical practice. Waiting times. Survival, recurrence rates/remaining tumour after resection and rate of metastasis.	Changes in diagnostic reports after TB discussion (between 4 and 35% of patients discussed at TBs); more likelihood to receive more accurate and complete pre-operative staging for patients discussed at TBs. Changes in patient management/clinical practice after discussion reported in 4.5–52% of cases. More likelihood to receive neoadjuvant/adjuvant treatment and greater adherence to guidelines for patients discussed at TBs. Limited evidence for improved waiting times. Limited evidence for improved survival of patients discussed at TBs; little positive impact on local recurrence rates/remaining tumour after resection and incidence of metastases.
Basta et al., 2017 [34] (The Netherlands)	PubMed, MEDLINE and EMBASE electronic databases. Until 30 November 2016.	16 (studies quality: fair)	Gastrointestinal malignancies: oesophageal or gastric, colorectal, pancreatic or biliary, liver malignancy or neuroendocrine, other malignancies.	To assess whether the discussion in a multidisciplinary gastrointestinal cancer team meeting influences the diagnosis and treatment plan for patients with GI malignancies.	Diagnosis and staging. Treatment plan and adherence to guidelines. Implementation of the treatment plan.	Changes in the diagnoses formulated by individual physicians after TB discussion (18.4–26.9% of evaluated cases); accurate diagnosis in 89–93.5% of cases evaluated by TBs; more frequent complete staging evaluation for patients discussed. Treatment plan altered in 23.0–41.7% of evaluated cases; increased adherence to guidelines. TB decisions implemented in 90–100% of evaluated cases.

The number of studies included in each review varied, ranging from 16 to 51 studies. Sixteen studies were included in the systematic review focused on lung cancer [33]; 16 in the review concerning gastrointestinal cancers [34]; and 51 [31], 27 [32] and 37 [30] studies were included in the other 3 reviews. Some of the articles included were found in two or more of the reviews considered. More precisely, one study was cited in four out of five reviews [30–32, 34] and three studies were shared by three reviews (one study in common by Prades [31], Pillay [32] and Basta [34]; one by Prades [31], Pillay [32] and Lamb [30] and one by Pillay [32], Basta [34] and Lamb [30]). Other primary studies were common to two reviews. In particular two reviews not focusing on a specific kind of tumor (Prades et al., 2015 [31] and Pillay et al., 2016 [32]) shared a higher number of articles, seven overall. Coory et al. [33] review was the only one that had articles in common only with Lamb et al. review [30]. However, 120 studies were considered globally.

The databases searched for the different reviews were PubMed, Ovid Medline, PsycINFO, Embase, and Cochrane. Two studies [31, 33] limited the research to one database and one of them also widened the research with a snowball search [33]. Two studies [32, 34] searched three databases each, while one [30] searched a total of four databases. Inclusion and exclusion criteria for each review were clearly stated and limitations were identified.

Although in general the descriptions of TBs were similar, the definitions reported by the five reviews showed a variability concerning the characteristics and composition of TBs both in terms of format and members (Table 2). On the contrary, all the reviews reported the same clinical objectives for the TBs, which was to discuss the diagnosis, treatment and management of oncological patients in a board composed of multiple healthcare professionals. The working format of TBs was described in a number of ways, but all the reviews agreed on the practice of holding scheduled meetings which could be either daily or weekly, in person, by video or teleconferencing. Only Prades et al., 2015 [31] cited TBs *Clinic* format in which patients were not only discussed but also simultaneously examined by all board members. Team members were determined based on factors such as type of cancer studied and hospital size. Therefore, each TB included different specialties of healthcare professional (surgeons, oncologists, pathologists, radiologists, nurses, etc.). In particular, Prades et al., 2015 [31] described the team according to three levels of involvement: core and allied members which are all medical specialists and support members that include both medical and non-medical professionals.

**Table 2:** TBs characteristics

	Coory et al., 2008 [33]	Lamb et al., 2011 [30]	Prades et al., 2015 [31]	Pillay et al., 2016 [32]	Basta et al., 2017 [34]
Definition	Team working among specialists with diagnostic and therapeutic intent, who meet to discuss the diagnosis and management of patients.	Group of different healthcare professionals, who meets together to discuss a patient. Each one is able to contribute independently to the diagnostic and treatment decisions about the patients.	Alliance of health care professionals related to a specific tumour disease. Approach to cancer care is guided by willingness to agree on evidence-based clinical decisions and to coordinate the delivery of care at all stages of the process, encouraging patients to take an active role in their care.	A regularly scheduled discussion of patients, comprising professionals from different specialties. The TB serves as a platform for the coordinated delivery of care through consultation amongst different professionals in a single setting.	Healthcare professionals from different medical specialties working together for specific diseases.
Intent	To discuss diagnosis, treatment and management of patients.	To discuss patients and contribute to the diagnostic and treatment decisions. To improve communication, coordination and decision-making between healthcare professionals.	Appropriate and up-to-date treatment, structured follow up plan. To improve coordination and continuity of care, to achieve early referral patterns.	Coordination of care within the team to ensure accurate staging, consideration of different treatment options, continuity of treatment, and follow-up.	To discuss and diagnose patients with complex diseases and formulate a treatment plan according to the guidelines.
Format	Meetings of specialists at specified time either in person, by video or teleconferencing.	Meetings of professionals at a given time, physically, by video or teleconferencing.	(1) Meetings of 30 min - 2 h including either all or a selection of diagnosed and/or referred patients. Patients selection by the specialist in charge on the basis of the case’s level of complexity or the wide range of therapeutic possibilities, prearranged team criteria, or triage by the clinical coordinator. (2) Clinics in which patients were seen and also simultaneously examined or remotely coordinated by all board members. (3) Online conferences within a given hospital or nationwide. Meeting presentations involving prospective reviews of new and recurrent cases, previously reviewed cases requiring additional follow-up, and second opinions.	TBs conducted either weekly or fortnightly (daily meetings reported in one study).	Team meetings at periodic intervals (i.e., daily or weekly).
Members	Thoracic physicians, thoracic surgeons, radiation oncologists, specialist radiologist, medical oncologists, pathologists, nursing and allied health staff and palliative-care specialists (there are different local configurations).	Several healthcare specialists.	Team members and attendance vary according to hospital size and medical specialty. Three levels of members involvement. (1) Core and (2) Allied: radiologists, pathologists, surgeons, radiation and medical oncologists, oncology nurses, palliative care physicians, head and neck specialists, nuclear medicine specialists, respiratory disease physicians, gastrointestinal disease physicians and anaesthesiologists; (3) Support: psychologists, nutritionists, dieticians, plastic surgeons, speech therapists, patients’ GPs, physiotherapists, practitioners of complementary medicine, orthopaedic specialists, medical physicists, odontologists, faith counsellors, biologists, data managers, genetic counsellors, hospital pharmacists, social workers and occupational therapists.	Surgeons, medical and radiation oncologists, radiologists, pathologists and nurse specialists. In addition, professionals from pharmacy, palliative medicine, mental health and other allied health disciplines may also be present.	Different medical specialists.

A structured quality assessment of the included papers was performed only in two systematic reviews (Basta et al., 2017 [34]; Lamb et al., 2011 [30]), using different tools. In Basta et al., 2017 [34] the ‘Quality Assessment Tool for Before–After (Pre–Post) Studies With No Control Group’ was used to assess the quality of before–after studies and the ‘Quality Assessment Tool for Observational Cohort and Cross-Sectional Studies’ was used to evaluate cohort studies. The papers were finally classified as fair. Also risk of bias was evaluated, using a single tool (‘To Assess the Risk of Bias in Cohort Studies’) for both study design. All studies scored as ‘moderate’ relating to risk of bias. In Lamb et al., 2011 [30] Authors chose an assessment tool to be used in systematic reviews with heterogeneous articles and the quality of papers was classified as low to medium [35]..

Quality assessment for the other reviews was performed in a non-structured manner (Coory et al. [33]) or not at all (Prades [31] et al. and Pillay [32] et al.).

### Outcomes and outputs

The outcomes and outputs reported by the reviews are presented in the following paragraphs. More details are shown in Table 3.

**Table 3:** Outcomes and outputs in the reviews retrieved. Main findings

Outcome / Output	Author / Year	N. of studies	Results / Findings
Care coordination	Prades et al., 2015 [31]	22	Format, data management and professional roles of TBs impacted positively on care coordination for professionals and patients.
Diagnosis (Patient assessment, diagnosis, staging)	Lamb et al., 2011 [30]	3	Improvement in diagnostic accuracy was reported.
	Prades et al., 2015 [31]	8	Multidisciplinary setting improved diagnosis and staging accuracy.
	Pillay et al., 2016 [32]	15	Diagnostic reports changed after the meeting in 4–35% of patients discussed.
		6	The impact of the TB on assessment and diagnosis was significant (higher accuracy in staging).
	Basta et al., 2017 [34]	1	No changes in diagnosis or stage were reported after validation by pathology or after follow-up.
		4	TBs changed the diagnoses formulated by referring physicians in 18.4–26.9% of cases.
		2	TBs formulated an accurate diagnosis in 89 and 93.5% of evaluated cases.
		2	Discussion during the TB influenced staging. After introduction of the TB, more patients underwent computed tomography (CT) before operation and patients discussed more often received a complete staging evaluation.
Treatment (Practice patterns, clinical practice, patient management, Implementation of treatment changes)	Coory et al., 2008 [33]	1	A not statistically significant larger percentage of patients discussed in TB (43%) received radical treatment than the control group (33%).
		1	A statistically significant increase in the percentage of patients older than 70 years receiving radical radiotherapy (from 3% in 1995 to 12% in 2000; *p* = 0.004) was reported. The percentage receiving palliative radiotherapy decreased (from 65 to 55%).
		1	A statistically significant increase in the percentage of patients receiving chemotherapy (from 7% in 1997 to 23% in 2001; *p* < 0.001) was reported. The percentage of patients receiving palliative care decreased (from 58 to 44%; *p* = 0.0045) and the percentage of patients being formally staged increased (from 70 to 81%; *p* = 0.035).
		3	Surgical resection rate was higher in MD groups.
	Lamb et al., 2011 [30]	6	Changes in care management decisions were reported in 2–52% of cases.
		1	TBs improved adherence to clinical guidelines.
		1	Likelihood of patients being offered chemotherapy increased (from 7 to 23%)
		6	Care management decisions by TBs were not implemented in 1–16% of cases due to contradictory patient choice or because of comorbidities.
	Prades et al., 2015 [31]	21	TBs ensured more appropriate treatment through preoperative review of imaging and pathology results; multidisciplinary approach guaranteed the most up-to-date treatment, and set up a structured follow-up care plan.
	Pillay et al., 2016 [32]	25	Changes in patient management/clinical practice were measured. Three studies reported minimal change in clinical management (less than 9% of cases), four studies indicated that the percentage of patients who underwent changes in treatment plans ranged from 19 to 34.5%. Other studies reported that changes in patient management plan following a TB occurred in 4.5–52% of cases.
		13	Patients who were discussed were more likely to receive neoadjuvant or adjuvant treatment. Greater adherence to National Comprehensive Cancer Network (NCCN) guidelines was found in two studies.
	Basta et al., 2017 [34]	9	Treatment plan formulated by the referring physician was altered in 23.0–41.7% of evaluated cases.
		5	TB decisions on treatment plan were implemented in 90–100% of evaluated cases. The reasons for not following TB advice were comorbidity (45%) and patient preferences (35%), followed by new clinical information (10%), different opinion of the treating physician (5%), and unknown (5%).
		3	TBs increased adherence to guidelines. Treatment plan more often adhered to national guidelines: 98% versus 83%.
Quality of life	Coory et al., 2008 [33]	1	No statistically significant difference between groups was found
	Prades et al., 2015 [31]	6	Improvement of patients’ quality of life
Recurrence and metastasis after resection	Pillay et al., 2016 [32]	2	TB discussion had little positive impact on local recurrence rates of rectal cancer and incidence of metastases and remaining pelvic tumour after resection.
Satisfaction (patient or clinician)	Coory et al., 2008 [33]	1	TBs resulted in better satisfaction for organisation of investigations and personal experience of care.
	Prades et al., 2015 [31]	5	TBs improved patient and clinician satisfaction as a consequence of team work communication and cooperation.
Survival	Coory et al., 2008 [33]	2	Two studies reported statistically significant survival improvement. 1 study reported an improvement of 3.2 months in median survival of patients with inoperable NSCLC, the other an increase from 18.3 to 23.5% in 1-year survival of lung cancer patients older than 70.
		3	Three studies did not show a statistically significant improvement.
	Lamb et al., 2011 [30]	1	Patients being offered chemotherapy showed a significant increase in survival (from 3.2 to 6.6 months).
	Prades et al., 2015 [31]	10	Improvements in survival were reported for colorectal, head and neck, breast, oesophageal, and lung cancer.
	Pillay et al., 2016 [32]	4	TB discussion was not associated with overall survival. However, in one of these studies, rectal cancer patients discussed had improved post-operative mortality.
		2	Significant association was shown between TB discussion and survival of patients.
Visits to general practitioners	Coory et al., 2008 [33]	1	Significantly fewer visits were reported for the MD group than the control group.
Waiting times	Coory et al., 2008 [33]	3	In one study the median time from presentation to first treatment was 3 weeks in the MD arm (7 weeks in the control arm) but there was no difference in the time from diagnosis to radical treatment. Another study reported a reduction in mean time from presentation to surgery of 15 days. In the last study, a reduction of days from diagnosis to treatment from 29.3 to 18.8 was reported.
	Prades et al., 2015 [31]	10	TBs resulted in reduction of time from diagnosis to treatment, and achievement of early and appropriate referral patterns.
	Pillay et al., 2016 [32]	2	In two studies patients discussed in TBs had fewer mean days from diagnosis to treatment.
		1	One study found an opposite trend.
Other	Prades et al., 2015 [31]	7	TBs promoted the establishment of a teaching environment for healthcare professionals and junior doctors.
		9	A commitment to research and clinical trials was maintained.
		1	The enrolment in the tumour registry increased.

#### Diagnosis

Four out of five reviews focused on different aspects of the diagnostic process. Eight studies examined by Prades et al., 2015 [31] suggested an improvement in diagnosis and staging accuracy promoted by the establishment of a multidisciplinary setting. Three studies in Lamb et al., 2011 [30] reported that TBs improve diagnostic accuracy. Fifteen studies by Pillay et al., 2016 [32] described changes in diagnostic reports after TB discussion and six studies comparing a TB group with a control group of patients saw a significant impact of TBs on patient assessment and diagnosis with higher accuracy in staging. Even Basta et al., 2017 [34] found that four studies showed a significant impact of TBs in changing the diagnosis formulated by the referring physicians, while one study described no changes in diagnosis or stage after validation by pathology or follow up. Two studies also evaluated the accuracy of the diagnosis itself, which was found to be accurate in 89.0 and 93.5% of cases. Finally, two studies found that there were influences in tumor staging related to TBs discussion.

#### Quality of life

Results concerning quality of life were described in two of the systematic reviews examined and they reported two different outputs. One study included in Coory et al., 2008 [33] found, by submitting a questionnaire to the patients, no-statistically significant differences between the patients examined in the TB and the control group, while six studies of Prades et al., 2015 [31] described an improvement on quality of life.

#### Recurrence and metastasis after resection

Only one review (Pillay et al., 2016) [32] reported the impact of TBs on recurrence of tumor and metastasis rates after surgical resection with two studies describing only a minimal positive impact on local recurrence rates of rectal cancer and incidence of metastases and remaining pelvic tumor after resection.

#### Survival

Results in terms of survival were also reported and showed non-homogeneous findings. Two studies from Coory et al., 2008 [33] reported statistically significant results while three studies did not show a statistically significant improvement. Ten studies from Prades et al., 2015 [31] described survival improvement for colorectal, head and neck, breast, esophageal, and lung cancer. Lastly, Pillay et al., 2016 [32] described both a significant association in two studies and no association in four studies, although one of these studies reported an improvement in post-operative mortality in rectal cancer patients discussed by TBs.

#### Treatment

All of the reviews retrieved highlighted an impact on treatment strategies and processes. Six studies described by Coory et al., 2008 [33] measured changes in treatment strategies. Prades et al., 2015 [31] focused on three aspects of treatment improvement which was enabled by the preoperative review of imaging and pathology results, the positive impact of the multidisciplinary approach regarding offering up-to-date therapies, and the ability to set up a structured follow-up plan. The review by Pillay et al., 2016 [32] also reported various degrees of changes in treatment management both in prospective and retrospective studies. Only three case-control studies found a non-significant difference in treatment. Basta et al., 2017 [34] described an alteration and enhancement of treatment plans with an increased adherence to guidelines. Finally, six studies included by Lamb et al., 2011 [30] reported changes in care management decisions, while other six studies described a lack of implementation of TB decisions. Moreover, in Lamb et al., 2011 [30] four studies reported that decisions were based on biomedical information while patient choice was considered infrequently. In fact, only a study in Lamb et al., 2011 [30] reported patient involvement in regard to the decision-making process revealing that the involvement was described only in 4% of the cases. Another result procured through this review was that four studies reported that telemedicine improves the attendance of meeting and allows to visualize pathological and radiological reports from different locations.

#### Care coordination

Only one review (Prades et al., 2015) [31] examined the impact of TBs format, data management and role of the professionals on care coordination for professionals and patients. The resulting impact was seen to be positive.

#### Satisfaction

Both patients’ and professionals’ satisfaction was described in some of the studies retrieved and improvement was noted for both cases. Patients’ satisfaction related to a better organization of investigations and to personal experience of care was described in one study included in the review by Coory et al., 2008 [33]; it was acquired through the submission of a questionnaire. Prades et al., 2015 [31] also found improved clinician satisfaction as a result of teamwork communication and cooperation.

#### Visits to general practitioners

Only one study, analysed by Coory et al., 2008 [33], highlighted the aspect pertaining to visits to General Practitioners (GPs). In this case, significantly fewer visits to GPs were reported for the TB group.

#### Waiting times

Prades et al., 2015 [31] reported an impact of TB interventions on the reduction of time from diagnosis to treatment and also on the achievement of early and appropriate referral patterns. Three studies included in Coory et al., 2008 [33] review also assessed this topic reporting a reduction of median time from presentation to first treatment, mean time from presentation to surgery and average number of days from diagnosis to treatment. Two studies included in Pillay et al., 2016 [32] described fewer mean days from diagnosis to treatment and one study demonstrated an opposite trend.

#### Other

The systematic review from Prades et al., 2015 [31] also described other areas of improvement promoted by TBs such as the establishment of a teaching environment for healthcare professionals and junior doctors, the increase in enrollment to tumor registries and stronger commitments to research and clinical trials.

### Quality assessment

According to the qualitative assessment tool AMSTAR2 scale, the five systematic reviews have been evaluated as “critically low” [29] because they presented more than one weakness among the critical domains previously described. Of the 16 domains assessed by the AMSTAR2 scale, all the reviews reported a negative answer in the domain concerning the source of funding reporting (10th domain). None of the reviews performed a meta-analysis (11th and 12th domains), nor an adequate investigation of publication bias with a discussion of its impact on the results (15th domain). Other weaknesses, although Basta et al., 2017 [34] got some partial positive responses, concerned the following domains: absence of a protocol provided before the conduct of the review (2nd domain) and absence of a defined technique to assess the risk of bias (9th domain).

## Discussion

TBs are recognized as an effective approach in cancer care to improve quality of healthcare processes and patient’s outcomes (Lamb et al., 2011) [30]. To our knowledge, this umbrella review is the first attempt to synthetize the vast amount of literature available concerning the impact of TBs on health outcomes and clinical processes. This study was performed to provide stronger evidence on this topic. In fact, the five reviews included in this paper collected information from 120 studies, although only qualitative and not quantitative syntheses of the results were provided.

Different aspects emerge from this paper to be considered for further discussion. Firstly, regarding the description of TBs, the findings of this umbrella review clearly confirm a progressive evolutionary trend - from 2008 to date - of TBs, whose characteristics have been changing and evolving over time. Initially created to provide a consultation by all physicians in charge at a specific point of the cancer treatment pathway [7], TBs have gradually acquired a more collaborative approach in which the decisions and clinical responsibility are shared by all the members of the TB, who also address all the aspects of treatment during all stages of the delivery of care. Patients could also be involved in the decision-making processes [7, 30–34].

Indeed, all five reviews consistently describe TB as the place where - although with format and members that can partly vary - health professionals discuss diagnosis, treatment and management of their patients in order to reach shared decisions, improve the diagnostic accuracy, get an accurate staging and provide the best evidence-based treatment and patients’ health outcomes [30–34]. Moreover, Lamb et al., 2011 [30], Prades et al., 2015 [31] and Pillay et al., 2016 [32] underline the fundamental role of TBs in improving communication, coordination and continuity of care among the different professionals involved in care process. Furthermore, the definition of TB by Prades et al., 2015 [31], includes encouraging patients to take an active role in their care.

Concerning the impact of TBs, the effects on diagnosis, treatment and survival were addressed by most of the reviews. Diagnosis was addressed by four reviews (Lamb et al., 2011 [30], Prades et al., 2015 [31], Pillay et al., 2016 [32], Basta et al., 2017 [34]), while treatment was discussed in all the reviews (Coory et al., 2008 [33], Lamb et al., 2011 [30], Prades et al., 2015 [31], Pillay et al., 2016 [32], Basta et al., 2017 [34]), and survival by four of them (Coory at al., 2008 [33], Lamb et al., 2011 [30], Prades et al., 2015 [31], Pillay et al., 2016 [32]).

In regard to diagnosis, improvements in the diagnostic and staging processes accuracy were reported (Lamb et al., 2011 [30], Prades et al., 2015 [31], Pillay et al., 2016 [32], Basta et al., 2017 [34]). In reference to treatments, the five reviews (Coory et al., 2008 [33], Lamb et al., 2011 [30], Prades et al., 2015 [31], Pillay et al., 2016 [32], Basta et al., 2017 [34]) showed that TB discussion led to changes in patient management/clinical practices. Pillay et al., 2016 [32] and Basta et al., 2017 [34] also reported an increased adherence to guidelines. Such impacts are attributable to the discussion during meetings which lead to modifications in both diagnostic paths and treatment plans in an effort to find the best alternatives for the patients while simultaneously adhering to clinical guidelines. Indeed, many guidelines specifically included the multidisciplinary approach as a tool for cancer patients management [15–17]. By offering different healthcare professionals the possibility to discuss cases while analyzing imaging and histological referrals, the TB facilitates multidisciplinary approach, that encourages and allows the exchange of knowledge. This collaboration among professionals with a variety of experiences allows for a more accurate diagnosis and assist practitioners to offer the best and most appropriate treatment (Basta et al., 2017 [34]; Lamb et al., 2011 [30]). Results on survival were not homogeneous. In some cases, as described by Prades et al., 2015, [31] survival improved, in others, as reported by Coory et al., 2008 [33], Lamb et al., 2011 [30] and Pillay et al., 2016 [32], evidence was limited. The lack of sufficient evidence on survival - as noted by Coory et al., 2008 [33] – may be attributed to a difficulty in conducting randomized clinical trials in order to demonstrate the potential impact of TB on its improvement, free from confounding factors. This last aspect is also addressed by Houssami & Sainsbury, 2006 [36], in which the survival benefit was shown to be related to the specialist who operated.

The other TB impacts resulting from this umbrella review were discussed by a fewer number of reviews or even by single studies, highlighting thence an important level of heterogeneity and a weaker evidence.

The reviews of Coory et al., 2008 [33] and Prades et al., 2015 [31] are the only two which investigated quality of life and patients’ satisfaction; two important goals to be pursued to provide a more holistic and personalized care approach. Related evidence is weak but promising, insinuating that these two aspects deserve further investigation (Prades et al., 2015) [31].

Other aspects (such as recurrence and metastasis after resection, care coordination, visits to GP and waiting times) were marginally reported in the retrieved reviews. Despite this, the results indicated a trend towards improvements in care, hence representing additional topics which deserve attention in future research and studies.

Although they may stray from the focus of this umbrella review, some points highlighted by the study are worth mentioning because they are relevant to the healthcare process. These topics are related to the lack of patient involvement in the decision-making process and a lower importance of the inputs provided by nurses during the meetings (Lamb et al., 2011) [30]. According to Pillay et al., 2016 [32], patient involvement in the decision-making processes could have an impact on the satisfaction level for care received as well as a more careful informed decision about treatment (Pillay et al. 2016) [32]. Moreover, as suggested by Lamb et al., 2011 [30], the position of nurses and other healthcare professionals should be reviewed and encouraged in the context of TBs. Given the nurses’ key role in the care process and coordination and their close relationship with the patient, reconsidering and strengthening the position and role of nurses in the TBs could enhance the implementation of treatment decisions improving patient’s outcomes (Lamb et al., 2011) [30].

Finally, sustainability factors for TBs should be assessed. As stated by Lamb et al., 2011 [30], telemedicine can represent a cost-effective way to increase the attendance to TBs in a cost-effective manner. According to Coory et al., 2008 [33], as a healthcare intervention, TBs should be evaluated by cost-effectiveness analysis, in order to estimate their clinical effectiveness in relation to their sustainability in the hospital setting (Coory et al., 2008) [33].

### Weakness and strengths

Despite the great number of studies considered (120) in the five reviews, a meta-analysis was not performed due to the high heterogeneity among all the studies. Relevant heterogeneity was also found among studies regarding the same cancer types, as reported by Basta et al. [34]. In fact, studies were referred to patients with different subtypes or at different stages of the same tumor. These two aspects can influence the assessment and management of patients (Pillay et al., 2016) [32]. Therefore, it would be better to investigate the impact of TBs on subgroups of patients. In the present study, results observed are thence related to cancer patients as a general group, as stated by Pillay and colleagues (2016) [32]. Moreover, heterogeneity among the studies is related to the different outcomes evaluated (Coory et al., 2008) [33].

Another weakness results from the variety of studies included in all the reviews. Most of them are observational studies, and therefore susceptible to bias because of the study design. This has probably influenced the methodological assessment, leading to a low-quality judgement. Moreover, surveys or qualitative studies are included in the reviews of Coory et al. (2008) [33], Lamb et al. (2011) [30], Prades et al. (2015) [31]. Although these reviews contain some experimental studies too, a final strong evidence was not observed; this is likely due to the heterogeneity among the studies within the same review.

Finally, another bias to consider is the potential publication bias. Since the Authors of the reviews only considered studies published in peer reviewed journals, it is possible that positive effects of TB may have been overestimated (Pillay et al., 2016 [32], Coory et al., 2008) [33].

Despite these limitations, the present work represents the first attempt to synthetize the research available on the topic of TBs and their impact on health outcomes and healthcare processes in a comprehensive manner and using a strict methodology. The only selection and inclusion of systematic reviews in our umbrella review was aimed at providing stronger evidence.

## Conclusion

### Recommendations for health professionals and academics

Definitively, TBs represent the best approach to a complex care pathway as cancer treatment because it improves decision making, patient care coordination, and it reduces waiting times. At the same time, its multidisciplinary feature is still challenging since it requires care coordination, effective decision-making, good communication, and the active participation of stakeholders including patients and all professionals. In order to improve the impact of TBs on healthcare delivery and health outcomes, the aforementioned factors should be addressed by healthcare managers to improve teamwork within their organizations [37]. Moreover, the difficulty professionals face in attending meetings, due to the lack of time, is a relevant barrier for the implementation of TBs (Coory et al. 2008) [33]. To make TBs effective, professionals should consider them a critical part of their working agenda and save time to prepare and attend TBs. Moreover, congruent time should be dedicated to the meetings in order to avoid discussing many cases in a short amount of time. Additionally, the time for acting on decisions made during the TBs should be taken into account (Lamb et al. 2011) [30]. To be addressed, all these points require a cultural change concerning the way clinicians and other health professionals understand their practice. Some changes have been made towards this and several of the latest guidelines recommend the MDT approach in order to provide better cancer care [15–17].

In order to assess the best way to functionally organize and deliver TBs and their cost-effectiveness, prospective studies should be carried out. Moreover, they could lead to better understand and control potential confounders while measuring impact in a more consistent way (Coory et. Al. 2008) [33].

For instance, although performing randomized studies can be time consuming and results have to be contextualized (Coory et al. 2008) [33], such studies could provide a solution to reduce bias and to gain more information. Moreover, health outcomes/outputs are not sufficient to evaluate the performance of TBs due to the complexity of these healthcare interventions. Thereafter, *[new]* process indicators, such as the ability to reach a decision on a first case or to implement decisions taken during TBs - as suggested by Lamb and colleagues [30] in their review - should be defined and measured in order to assess TBs in a more comprehensive and exhaustive way.

## Data Availability

Not applicable.

## Abbreviations

EPAAC: European Partnership for Action Against Cancer
GPs: General Practitioners
MDT: multidisciplinary team
NHS: National Health Service
TBs: Tumor Boards
